# Gamma-Ray Attenuation to Evaluate Soil Porosity: An Analysis of Methods

**DOI:** 10.1155/2014/723041

**Published:** 2014-01-29

**Authors:** Luiz F. Pires, André B. Pereira

**Affiliations:** ^1^Department of Physics, State University of Ponta Grossa (UEPG), Avenue Carlos Cavalcanti 4748, 84.030-900 Ponta Grossa, PR, Brazil; ^2^Department of Soil Science, State University of Ponta Grossa (UEPG), Avenue Carlos Cavalcanti 4748, 84.030-900 Ponta Grossa, PR, Brazil

## Abstract

Soil porosity (*ϕ*) is of a great deal for environmental studies due to the fact that water infiltrates and suffers redistribution in the soil pore space. Many physical and biochemical processes related to environmental quality occur in the soil porous system. Representative determinations of *ϕ* are necessary due to the importance of this physical property in several fields of natural sciences. In the current work, two methods to evaluate *ϕ* were analyzed by means of gamma-ray attenuation technique. The first method uses the soil attenuation approach through dry soil and saturated samples, whereas the second one utilizes the same approach but taking into account dry soil samples to assess soil bulk density and soil particle density to determine *ϕ*. The results obtained point out a good correlation between both methods. However, when *ϕ* is obtained through soil water content at saturation and a 4 mm collimator is used to collimate the gamma-ray beam the first method also shows good correlations with the traditional one.

## 1. Introduction

Soil porosity (*ϕ*), represented by the ratio of the pore volume to the total volume of a representative sample, is the functional entity of soil structure. Its value in soil generally varies from 0.3 to 0.6 cm^3 ^cm^−3^. This soil physical property is of fundamental importance for environmental studies due to the fact that water infiltrates and suffers redistribution in the soil pore space. Changes in *ϕ* directly affect the size, distribution, and the continuity of pores [1].

Many physical and biochemical processes related to environmental quality occur in the soil porous space. Porosity is also linked to the root growth and movement of air, water, and solutes in the soil. Well-structured soils are substantially important to maintain an adequate water transmission and gas exchange so that the development of the root system might be established for crop production with environmental protection at a given site. Soils with coarse textures tend to present less porous spaces than the fine ones [2].

Traditionally *ϕ* can be determined by measuring the soil water content at saturation or from the bulk and particle density relationship [3]. However, there are other methods based on nuclear techniques, such as gamma-ray attenuation (GRA) or computed tomography (CT) [4, 5]. The main objective of traditional or nuclear methodologies is to obtain representative values of *ϕ*.

GRA and CT are based on the interaction of radiation with matter. The knowledge of these interactions is important to understand X- or gamma-ray detection and attenuation processes. When a gamma-ray beam of incident intensity *I*_0_ (cps) interacts with a soil of thickness *x*, the transmitted intensity *I* (cps) through the absorber follows the Beer-Lambert law:

(1) $$\begin{array}{c} I=I_{0}e^{-\kappa x}. \end{array}$$

The term *κ* presented in (1) is the linear attenuation coefficient that measures the probability per unit length of a photon to be absorbed or scattered while interacting with a sample. *κ* represents the sum of several individual attenuation coefficients mainly due to the photoelectric absorption, Compton scattering, and pair production [6]. Owing to the fact that *κ* is dependent on the physical density *ρ* of the material, usually such a property is divided by *ρ* in order to obtain the mass attenuation coefficient *μ*. *μ* is almost independent of the physical state of the material.

It is important to mention that one of the greatest advantages of nuclear methods over traditional ones for measuring *ϕ* is the nondestructive analysis of the material structure. Such methods are fast and provide evaluations with a high spatial resolution (*μ*m to mm). Traditionally the most common radioactive sources used in soil physical analysis are the ^241^Am (*≈*60 keV) and ^137^Cs (*≈*662 keV) [7–10].

The main aim of the current research article is to present an analysis of methods proposed to measure soil porosity by means of the gamma-ray attenuation technique. Soil porosity was obtained via assessments performed on the mass attenuation coefficient determined both experimentally and theoretically (XCOM). The results of the nuclear method were compared to the traditional approach in study.

## 2. Experimental Details

### 2.1. Soil Samples

Soil samples with two different soil textures were analyzed. Disturbed samples were collected from an experimental field belonging to the University of São Paulo—ESALQ/USP—located at Piracicaba, SP, Brazil (22°42′S e 47°38′W, 580 m above sea level). The first set of soil samples was classified as a sandy loam and the second one as clay [1].

For *κ* and *μ* evaluations, samples were dried in oven at 105°C (48 h) and sieved through a 1 mm mesh sieve aiming to obtain a more homogeneous sample. Throughout the measurements the samples were kept in containers with silica-gel to prevent water from being absorbed from the environment.

### 2.2. Elemental Analysis

Semiquantitative elemental analysis of the soils was accomplished by energy dispersive XRF by using the instrument model EDX-720 (Shimadzu) equipped with an Rh X-ray tube. The equipment voltage varies from 5 to 50 kV and its tube current from 1 to 1000 *μ*A. The system detector is a Si(Li) semiconductor cooled with liquid N at −196°C.

An aliquot (2 g) of finely ground soil was then placed in a sample analysis cup (supplied by the equipment manufacturer) for measurements. The sample cup was covered with a Mylar film (6 *μ*m thickness) for analysis. The measuring time for each sample was 100 s in the energy region of Na-Sc with a voltage of 15 kV and 100 s in the energy region of Ti-U with a voltage of 50 kV. The measurements were performed under a pressure of 30 Pa. More details about the equipment usage procedures might be found at Shimadzu [11].

### 2.3. Mass Attenuation Measurement

The radioactive sources of ^241^Am (7.4 GBq) and ^137^Cs (11.1 GBq) were used in this study. The detector was a 7.62 × 7.62 cm NaI(Tl) scintillation crystal coupled to a photomultiplier tube. Circular lead collimators were adjusted and aligned between source (2 mm diameter) and detector (4.5 mm diameter) in order to produce a narrow beam. During measurements the criterion *κx* < 1 was used in order to minimize the number of multiple scattered photons reaching the detector [12].

Acrylic containers having 0.5 cm thick edge were filled with soil for *μ* measurements. The dimensions of the containers were 7.03 × 6.50 × 4.03 cm^3^ (^241^Am) and 7.03 × 6.51 × 8.04 cm^3^ (^137^Cs). The symmetry axis of the gamma-ray equipment arrangement was a horizontal line adjusted by a laser beam. Radiation spectra were evaluated before experimental *μ* determination, which made it possible to adjust the photopeak windows throughout the measurement periods. A 2 mm collimator and counting times of 30 s (^137^Cs) and 60 s (^241^Am) were used in the spectra measurements without sample (free beam). The radioactive source and detector were mounted 18.0 cm apart and the acrylic box containing the samples was centered and aligned between them.

**Figure 1:**
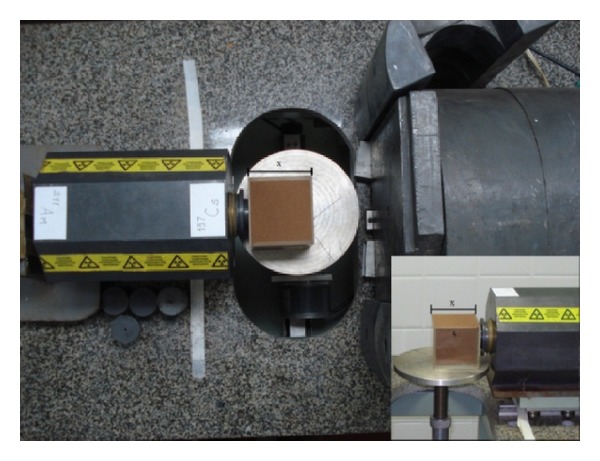
Photograph of the gamma-ray attenuation equipment [13].

The acrylic box was placed close to the source exit, touching the collimator, so that the beam could go through it as close as possible to the center of the sample and perpendicularly to this position in order to guarantee that the gamma-ray beam passes through the thickness (*x*) measured (Figure 1). The laboratory temperature was kept constant at 21 ± 1°C. The intensities of monoenergetic photons were measured at one unique position in the center of the acrylic box filled with soil. The soil mass attenuation coefficients determined herein depict only one measurement for each soil type sampled. Counting times adopted in *μ* measurements were 600 s (^137^Cs) and 900 s (^241^Am), respectively. The very same experimental setup was used for the measurement of water *μ*.

The following equations are utilized to obtain *μ* and its respective experimental error (*σ*_*μ*_):

(2) μs,w=1x·ρs,w·ln⁡(I0I),σ2μs,w=(∂μs,w∂x)2·σ2x+(∂μs,w∂ρs,w)2·σ2ρs,w +(∂μs,w∂I0)2·σ2I0+(∂μs,w∂I)2·σ2I,

where *ρ*_*s*,*w*_ (g cm^−3^) is the soil or water physical densities and *μ*_*s*,*w*_ (cm^2^ g^−1^) is the soil and water mass attenuation coefficients.

The theoretical *μ* was evaluated by using the software XCOM [14]. The XCOM is a computer code developed to calculate X-ray and gamma-ray attenuation coefficients and interaction cross sections for pure elements (*Z* = 1–100), compound or mixture at a wide energy range (1 keV–100 GeV). With the help of this software it is also possible to obtain partial cross sections for photoelectric absorption, scattering (incoherent and coherent), and pair production.

### 2.4. Porosity Measurement

By using the traditional method, *ϕ* (cm^3 ^cm^−3^) was indirectly assessed by means of the determination of soil bulk and particle densities [3]:

(3) $$\begin{array}{c} \phi_{1}=\left(1-\frac{\rho_{s}}{\rho_{p}}\right), \end{array}$$

where *ρ*_*p*_ and *ρ*_*s*_ (g cm^−3^) are the soil particle and bulk densities.

Total porosity obtained by means of the gamma-ray attenuation approach was evaluated taking into consideration two distinct methodologies. The first method is related to the equation proposed by Baytaş and Akbal [15] (4), whereas the second method is based on the determination of *ρ*_*s*_ through (1), written for the case of a soil as a porous material [16], and substitution of its value in (3) in order to come up with (5):

(4) $$\begin{array}{c} \phi_{2}={\left(1+\frac{x_{s}\kappa_{w}}{\ln(I_{\text{ds}}/I_{\text{ss}})}\right)}^{-1}, \end{array}$$

(5) $$\begin{array}{c} \phi_{3}=\left(1-\frac{1}{x\mu_{s}\rho_{p}}\ln\left(\frac{I_{0}}{I_{\text{ds}}}\right)\right), \end{array}$$

where *x*_*s*_ (cm) is the solid thickness, *κ*_*w*_ (cm^−1^) is the linear attenuation coefficient of water, and *I*_ds_ and *I*_ss_ represent the beam intensity after having passed through the box filled with dry soil and saturated soil. The other variables (*I*_0_, *x*, *μ*_*s*_,  and  *ρ*_*p*_) were previously described.

**Figure 2:**
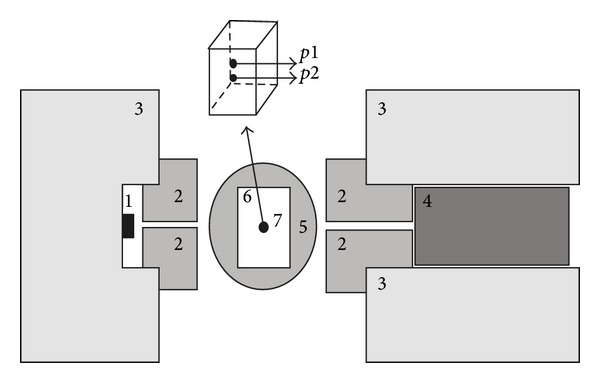
Schematic diagram of the gamma-ray attenuation equipment. (1) Radioactive source; (2) Pb circular collimators; (3) Pb shields; (4) NaI(Tl) detector; (5) table of measurement; (6) sample; (7) position (*p*) of the experimental measurements.

In the experimental procedures by using the nuclear method the intensities of monoenergetic photons were measured in two different positions along the center of the acrylic box filled with soil (Figure 2). For the measurement of *ϕ*, the circular lead collimator at source exit used previously for *μ* evaluation (2 mm diameter) was replaced with one 3 mm diameter collimator. In order to measure *ϕ* and *μ* it is important to highlight that different soil samples were taken into account and, therefore, different geometries in terms of collimators were used herein. The thickness (*x*) of the samples in the way of the beam was 4.03 cm (^241^Am) and 8.04 cm (^137^Cs), respectively.

The acrylic box was placed close to the source exit, touching the collimator, so that the beam could go through it as close as possible to the center of the sample and perpendicularly to this position as previously mentioned. The laboratory temperature was kept constant at 21 ± 1°C. Counting times adopted in *ϕ* measurements were 300 s for both sources.

The monitored background radiation was *≈*6974 cps. The uncertainties ($\sqrt{I}$) due to the statistic emission of a photon by the radioactive sources for *I*_0_ were 0.06% (^137^Cs) and 0.18% (^241^Am). Regarding *I*_ds_ and *I*_ss_ the uncertainties were 0.09% and 0.10% (^137^Cs) for both soils and 0.43% and 0.49% (^241^Am) for sandy and 0.51% and 0.62% (^241^Am) for clayey soils.

In order to compare theoretical and experimental results of *ϕ* among methods relative differences (RD) were calculated using the following equation:

(6) $$\begin{array}{c} \text{RD}=\left(\frac{\phi_{\text{trad}}-\phi_{\text{gra}}}{\phi_{\text{trad}}}\right)\cdot 100\%, \end{array}$$

where trad and gra are related to the traditional and nuclear methodologies.

## 3. Results and Discussion

### 3.1. Elemental Analysis

The XRF technique represents a strong analytical tool that allows fast, noninvasive, and accurate analyses of elemental composition of solid and liquid samples. Weight concentrations of elements in oxide form for the two soil textures analyzed are shown in Table 1.

The analysis of the data presented in Table 1 reveals that over 95% of the sandy soil is due to SiO_2_, Al_2_O_3_, and Fe_2_O_3_, while for the clayey soil SiO_2_, Al_2_O_3_, Fe_2_O_3_, and TiO_2_ were the most significant elements in different proportions under the experimental conditions.

### 3.2. Mass Attenuation Coefficients

Through the elemental analysis, it was possible to perform theoretical evaluations of *μ* for both soils by means of the XCOM computer code.  Table 2 illustrates the calculated and experimental values of *μ* of the soils in study.

Results obtained demonstrate that for low energy photons, as it is the case of ^241^Am, the clayey soil has a higher total attenuation of radiation in relation to the sandy one (RD of +26.7%), whereas for the medium energy photons, as it is the case of ^137^Cs, the outcomes lead to a slight inversion (RD of −1.6%). One explanation for such a differential response is the higher amount of Fe_2_O_3_ in the clayey soil (relative difference +79.3%) under low energies [17]. For energy values corresponding to that found in the ^137^Cs source, the differences in the chemical composition of the two soils evince little interference in the *μ* values.

**Figure 3:**
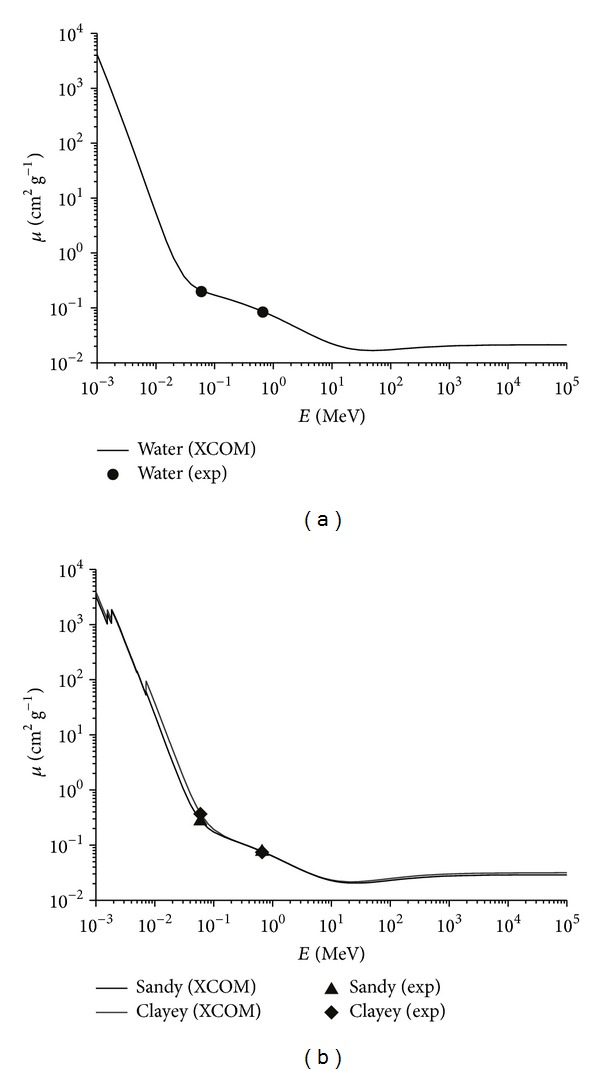
Experimental (Exp) and calculated (XCOM) mass attenuation coefficient (*μ*) variation as a function of photon energy (*E*). Experimental data for water (a) and sandy and clayey soils (b) were obtained for photon energies of *≈*60 keV (^241^Am) and *≈*662 keV (^137^Cs).

The comparison between calculated and measured results of *μ* shows RD of +4.0% (^241^Am) and +2.0% (^137^Cs) for water and +3.9% (^241^Am) and +1.6% (^137^Cs) for sandy and +2.2% (^241^Am) and +2.6% (^137^Cs) for clayey soils. The values of this variable are in agreement with those obtained by Ferraz and Mansell [18] for the same type of soils. Small discrepancies concerning the research published by the aforementioned authors are ascribed to differences in soil texture [1]. Graphs of *μ* variation for water and soils calculated by the XCOM program as a function of the radiation energy (1 keV to 100 MeV) are shown in Figure 3. This energy range comprises the two most used energies in soil physical properties evaluations through GRA technique.

Regarding the analysis of *μ* variation as a function of photon energy, the Rayleigh scattering and Photoelectric effect present *Z* ^2-3^ and *Z* ^4-5^ dependences and the energy region where the processes are dominant is <30 keV. For Compton scattering the *Z* dependence is linear and the dominant energy region varies within the range between 150 keV and 3 MeV. Pair production in nuclear and electron fields has *Z* ^2^ and *Z* dependences and dominant energy regions >50 MeV [19]. Such a *Z* dependence reflects the probability of occurrence of the effects aforementioned [6].

By analyzing the calculated *μ* variation with the coherent scattering (Figure 3(b)) it was possible to verify some slight differences between the two soils, which remained constant throughout the whole energy spectrum taken into consideration in the current study. A similar behavior was observed regarding the photoelectric effect. In the photoelectric effect region, a good agreement between measured and calculated *μ* results for both soils was obtained. Regarding the incoherent scattering (Figure 3(b)), there were not significant differences between the soils, which were already expected to a certain degree, since this effect shows linear dependence on *Z*. Concerning the differences observed between the two soils in the low energy region, such discrepancies might be explained mainly by the significant difference of Fe_2_O_3_ as discussed previously.

### 3.3. Soil Porosity

Some physical attributes of the soils used to evaluate *ϕ* by the traditional and nuclear methods are presented in Table 3.

**Figure 4:**
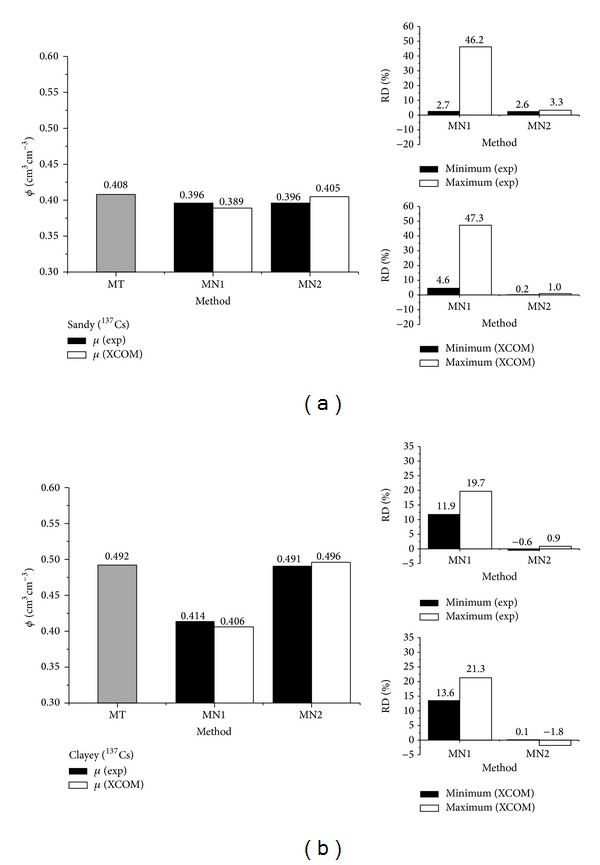
Soil porosity (*ϕ*) determination by using the traditional (MT) (3) and gamma-ray attenuation (MN1 (4) and MN2 (5)) methods for the ^137^Cs gamma-ray source for the sandy (a) and clayey (b) soils. RD represents the relative difference between the results of MT and MN (6). Porosities were calculated considering experimental (exp) and calculated (XCOM) mass attenuation coefficients (*μ*). Maximum and minimum values represent RD obtained from the different positions (*p*1-black box and *p*2-white box) of soil sample crossed by the gamma-ray beam.

**Figure 5:**
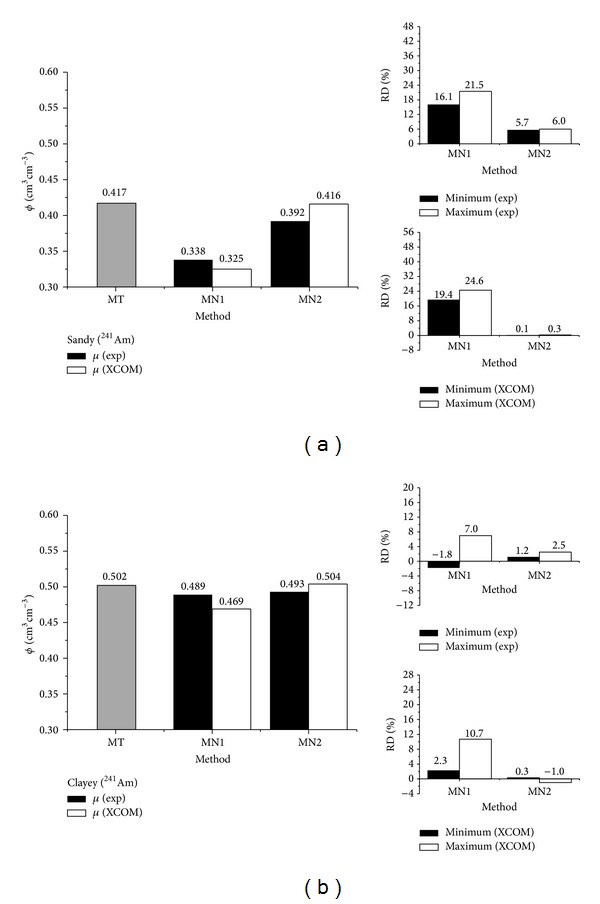
Soil porosity (*ϕ*) determination by using the traditional (MT) (3) and gamma-ray attenuation (MN1 (4) and MN2 (5)) methods for the ^241^Am gamma-ray source for the sandy (a) and clayey (b) soils. RD represents the relative difference between the results of MT and MN (6). Porosities were calculated considering experimental (exp) and calculated (XCOM) mass attenuation coefficients (*μ*). Maximum and minimum values represent RD obtained from the different positions (*p*1-black box and *p*2-white box) of soil sample crossed by the gamma-ray beam.

The similarities observed between the values of *ρ*_*s*_ for the two radioactive sources are an indication of the homogeneity in the procedure used to fill the acrylic boxes with the soils. Such response is important in order to compare the results of *ϕ* between radioactive sources. The outcomes of *ϕ* evaluated by the traditional (MT) and nuclear (MN) methods for ^137^Cs and ^241^Am gamma-ray sources and soils are demonstrated in Figures 4 and 5. In order to clarify the discussion of the outcomes MN methods will be divided into MN1 and MN2, which depict *ϕ* measurements by means of (4) and (5), respectively.

The responsiveness for the sandy soil and radioactive source of ^137^Cs (Figure 4(a)) shows a regular to a good correlation between MNs and MT. The minimum RD obtained by using the experimental *μ* corroborates this result. The minimum and maximum RDs are related to the position of the sample scanned (*p*1 or *p*2) (Figure 2). The choice of *p*1 and *p*2 was based on the experiment set up by Baytaş and Akbal [15]. Moreover, these two points of analysis were also selected to exam the effect of soil filling into the box and its impact on the determination of *x*_*s*_.

The absolute value of RD and their average values considering the experimental *μ* were 24.5% (MN1) and 2.9% (MN2), respectively. Taking into account the calculated *μ* (XCOM), the minimum and maximum RDs are almost similar to those obtained for the experimental *μ* for MN1 whilst the performance for MN2 has been improved (absolute average RD of 0.6%). Such a result corroborates the quality of the elemental analysis and confirms the good correlation observed between theoretical and experimental calculations of *μ* as shown in Figure 3 [20–22].

In the current study the values of *ϕ* determined by means of MT and MN1 and MN2 [15, 23] presented practically the same tendency in relation to the outcomes obtained by Medhat [24], that is, *ϕ* larger for MT than MNs. Large differences of *ϕ* observed between MT and MN1 can be explained by the variability of *x*_*w*_ and *x*_*s*_ among points of measurement. Usually clayey soils have a proclivity to form small aggregates after meshing [1–3], which tend to affect the homogeneity of the sample filled in the acrylic box.

The variations for the clayey soil (Figure 4(b)) show similar results in comparison to the sandy one. Minimum and maximum RDs reveal a small variability in *ϕ* measurement among points for MN2 again. Considering the absolute value of RD, their average values for the experimental *μ* were of 15.8% (MN1) and 0.7% (MN2) and the calculated values of *μ* were of 17.4% (MN1) and 0.9% (MN2), respectively.

On the other hand, if the value of *ϕ* for MT is to be considered as *θ* at saturation (Table 3), the average RDs (minimum and maximum) for MN1 will be −19.0% and 34.2% for the sandy and −3.3% and 5.9% for the clayey soils, respectively. These results were obtained for the experimental *μ*. In the case of calculated *μ* the results will be of −16.7% and 35.5% for the sandy and −1.2% and 7.7% for the clayey soils, respectively. The use of *θ*_*s*_ to obtain *ϕ* seems to show a better correlation with MN1 as opposed to MN2 in comparison to MT. Such behavior is not staggering at all because *ϕ* determined from *θ*_*s*_ leads to smaller values than *ϕ* evaluated from the bulk and particle density relationship [1]. This occurs mainly due to the entrapped air during the procedure of soil saturation [25]. Once in the current work the saturation was carried out with water being pounded at the surface of the sample, the presence of entrapped air is inevitable.

The results for sandy and radioactive source of ^241^Am (Figure 5(a)) show again a regular to a good correlation between MT and MNs. Considering the absolute value of RD, their averages in relation to the experimental *μ* were 18.8% (MN1) and 5.8% (MN2), respectively. For the calculated *μ* (XCOM) the minimum and maximum RDs are almost similar to those obtained for the experimental *μ* for MN1, while the performance of MN2 was significantly improved (absolute average RD of 0.2%).

For the clayey soil (Figure 5(b)) the correlation between MT and MNs might be seen as good to very good. As to the absolute value of RD, their averages in relation to the experimental *μ* were of 4.4% (MN1) and 1.8% (MN2), respectively. For the calculated *μ* (XCOM) the minimum and maximum RDs are almost similar to those obtained for the experimental *μ* for MN1 (absolute average RD of 6.5%), whereas for MN2 the accuracy was improved over again (absolute average RD of 0.7%).

The worst RD value for MN1 for both soils taking into consideration XCOM data is associated with the large *μ* obtained via elemental analysis that affects the calculation of *ϕ* by means of (4). In relation to MN2 the improvement of *ϕ* for the calculated *μ* is attributed to likely experimental errors during *κ* evaluation process for small energy gamma-ray photons [18, 26, 27].

On the other hand, if the value of *ϕ* for MT is considered to be *θ* at saturation (Table 3) the average RDs (minimum and maximum) for MN1 will be −2.9% and −10.0% for the sandy and −14.2% and −24.9% for the clayey soils, respectively. These results were obtained for the experimental *μ*. In the case of calculated *μ* the outcomes will be 1.3% and −5.6% for the sandy and −9.6% and 19.9% for the clayey soils, respectively. The use of *θ* at saturation to obtain *ϕ* seems to be conducive to a better correlation with MN1 rather than with MN2 in comparison to MT.

**Figure 6:**
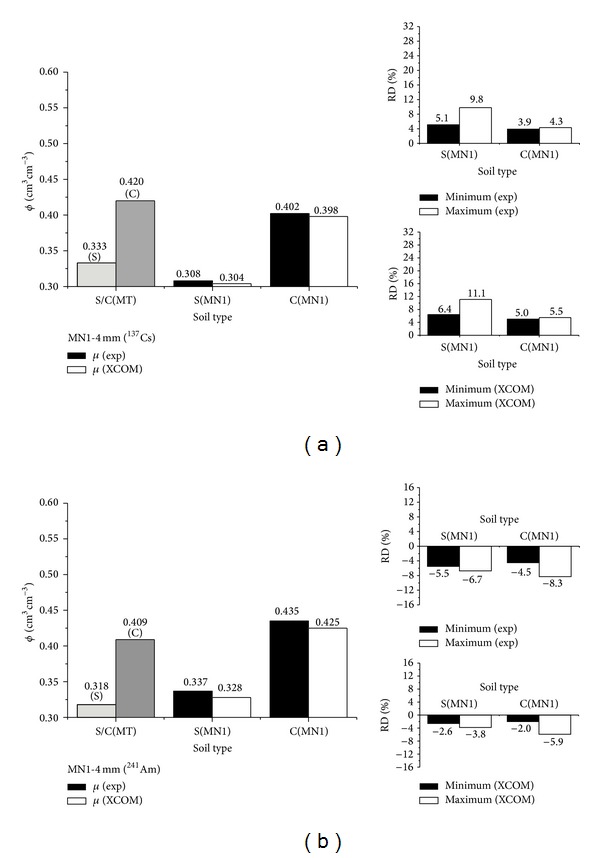
Soil porosity (*ϕ*) determination by using the traditional (MT) (3) and gamma-ray attenuation (MN1 (4)) methods for the ^137^Cs and ^241^Am gamma-ray sources and both soils (sandy-S and clayey-C). RD represents the relative difference between the results of MT and MN (6). Porosities were calculated considering experimental (exp) and calculated (XCOM) mass attenuation coefficients (*μ*). Maximum and minimum values represent RD obtained from the different positions (*p*1-black box and *p*2-white box) of soil sample crossed by the gamma-ray beam.

Moreover in Figure 6 the bottom line of *ϕ* is obtained for the 4 mm collimator inserted in the exit of the gamma-ray source. The choice of this collimator size is due to the fact that it can access a larger volume of water. The objective of such analyses was to show the effect of the collimator size increase on the sensibility of MN1 to evaluate the total porosity. For this, *ϕ* values were compared with those obtained via *θ*_*s*_ (Table 3).

It is possible from the upshots pointed out by Figure 6 to observe a better correlation between MT and MN1 in relation to what can be visualized in Figures 4 and 5. The scenario observed is mainly due to the increase in the soil volume in the same fashion of the gamma-ray beam technique [28]. Perhaps, the results of MN1 might be bettered by using collimators with large size. However, in order to conjecture about this it is necessary to pay attention to the question related to a good geometry [10, 27, 29, 30]. Considering the absolute value of RD, their averages with regard to the experimental *μ* were 7.5% (sandy) and 4.1% (clayey) for ^137^Cs and 6.1% (sandy) and 6.4% (clayey) for ^241^Am. For the calculated *μ* (XCOM) the obtained results were 8.8% (sandy) and 5.2% (clayey) for ^137^Cs and 3.2% (sandy) and 3.9% (clayey) for ^241^Am.

A comparison of *ϕ* results between gamma-ray sources demonstrates a good correlation between MT and MNs for the ^241^Am in relation to ^137^Cs gamma-ray source mainly for the XCOM data. The outcome in question is linked to the greater sensibility of photon interaction with water and soil structure for this gamma-ray energy [18, 31]. However, in general the results are quite good between gamma-ray sources especially for MN2. It is necessary to bear in mind that all results of *ϕ* obtained in this work (Figures 4 and 5) were used as a reference point for the traditional method described in (3). The pore volume can, in principle, be evaluated directly by measuring the volume of water needed to completely saturate the sample (Table 3 and Figure 6). In practice, however, it is always difficult to saturate the soil sample completely and, therefore, the total porosity of the sample turns out to be seldom evaluated by such a specific direct method [1–3].

## 4. Conclusions

In this work the gamma-ray attenuation technique is used for the evaluation of soil porosity by using two different methods: first one uses the soil attenuation through the dry soil and through saturate samples and the second one utilizes the attenuation through the dry soil to evaluate soil bulk density and by using the soil particle density its soil porosity is measured.

Soil porosity through the traditional method presents better correlation with the second method in relation to the first one. However, when soil porosity is determined through soil water content at saturation and a collimator of 4 mm is used to collimate the gamma-ray beam the first method also presents good correlations with the traditional one.

## Figures and Tables

**Table 1:** The chemical composition of soils.

Soil	Chemical components (weight)							
	SiO_2_	Al_2_O_3_	Fe_2_O_3_	TiO_2_	SO_3_	CaO	MnO	Others
Sandy	627.95	300.32	34.69	19.25	13.85	2.27	0.31	1.36
Clayey	441.87	328.39	167.37	34.86	17.12	1.55	3.23	5.61

**Table 2:** Calculated (XCOM) and experimental mass attenuation coefficients (*μ*) of the soils studied and water.

Sample	*μ* (cm^2^ g^−1^)			
	XCOM		Experimental	
	^ 241^Am	^ 137^Cs	^ 241^Am	^ 137^Cs
Water	0.2066	0.0857	0.1983	0.0840
Sandy (soil)	0.2807	0.0767	0.2698	0.0755
Clayey (soil)	0.3764	0.0763	0.3682	0.0743

**Table 3:** Soil physical properties used in the calculations of soil porosity (*ϕ*).

Soil	Characteristics			
	*ρ*_*s*_ (g cm^−3^)*	*ρ*_*p*_ (g cm^−3^)*	*U*_*s*_ (g g^−1^)*	*θ*_*s*_ (cm^3^ cm^−3^)*
	^ 137^Cs			
Sandy	1.51	2.55	0.221	0.333
Clayey	1.36	2.68	0.308	0.420

Soil	Characteristics			
	*ρ*_*s*_ (g cm^−3^)*	*ρ*_*p*_ (g cm^−3^)*	*U*_*s*_ (g g^−1^)*	*θ*_*s*_ (cm^3^ cm^−3^)*

	^ 241^Am			
Sandy	1.49	2.55	0.214	0.318
Clayey	1.33	2.68	0.307	0.409

The variables *ρ*_*s*_, *ρ*_*p*_, *U*_*s*_ and *θ*_*s*_ represent the soil bulk and particle densities and gravimetric and volumetric soil water contents.
